# Physiological and Transcriptomic Analyses Revealed That Humic Acids Improve Low-Temperature Stress Tolerance in Zucchini (*Cucurbita pepo* L.) Seedlings

**DOI:** 10.3390/plants12030548

**Published:** 2023-01-25

**Authors:** Haiping Li, Fanrong Kong, Tingting Tang, Yalan Luo, Haoran Gao, Jin Xu, Guoming Xing, Lingzhi Li

**Affiliations:** College of Horticulture, Shanxi Agricultural University, Jinzhong 030801, China

**Keywords:** humic acid, low temperature stress, transcriptomic analysis, metabolism reprogramming, antioxidative defense, zucchini

## Abstract

Zucchini (*Cucurbita pepo* L.) is one of the main vegetable crops grown under protected cultivation in northern China. Low-temperature (LT) stress severely inhibits the growth of zucchini seedlings, resulting in reductions in yield and quality. Here, using three kinds of different humic acids, including coal-based humic acid (CHA), fulvic acid (FA), and biochemical humic acid (BHA), we investigated the effects of humic acids against LT stress (5 °C) in zucchini seedlings. Treatment with all three kinds of humic acids improves LT stress tolerance by decreasing oxidative damage through increases in antioxidative enzyme activities and the contents of soluble sugar and proline in zucchini seedlings, especially after BHA application. Comparative transcriptomic analysis revealed that a total of 17 differentially expressed genes (DEGs) were commonly induced in the leaves of FA-, CHA-, and BHA-treated zucchini seedlings under LT stress, including calmodulin, ethylene-responsive transcription factors (TFs), peroxidases, and 10 TFs, including two *NAC* and seven *WRKY* genes. Altogether, these results indicated that supplementation with humic acids reprograms plant metabolism and modulates the expression of genes involved in ROS scavenging, phytohormone metabolism, or signaling pathways, finally improving LT stress tolerance in zucchini seedlings.

## 1. Introduction

Low-temperature (LT) stress includes chilling stress and freezing injury in plants [1]. The inhibitory effects of LT stress on plant growth and development run through the whole plant life cycle, such as seed germination, growth, photosynthesis, and fruit ripening [2]. LT stress leads to leaf etiolation, necrosis, changes in morphological characteristics, and a decrease in photosynthetic efficiency, finally resulting in a reduction in yield and quality [2]. LT stress disrupts cell membrane structure and influences cell membrane stability, thereby resulting in oxidative damage in plants [3,4]. Calcium (Ca^2+^) signaling and phytohormone signaling are important signal transduction pathways for plants in response to LT stress [5,6,7,8]. Antioxidative enzymes, such as superoxide dismutase (SOD), peroxidase (POD), and catalase (CAT), and antioxidant substances, such as glutathione (GSH) and ascorbic acid, can balance the production and scavenging of intracellular reactive oxygen species (ROS) and inhibit the peroxidation of unsaturated fatty acids in cell membranes, thus protecting the cell membrane and improving stress tolerance in plants [9,10]. In recent years, the physiological and molecular mechanisms underlying plant responses to LT stress have been widely investigated [5,11]. C-Repeat Binding Factors (CBFs) are core transcription factors (TFs) in plants involved in the response to LT [5,11]. Inducer of CBF expression 1 (ICE1) modulates LT stress adaptation by activating *CBF* gene expression [12]. Therefore, the ICE1-CBF transcriptional network plays a critical role in cold acclimation in plants. In addition to ICE1, other TFs that directly regulate *CBF* genes include the positive regulator Calmodulin-Binding Transcription Activators (CAMTAs) and negative regulators such as PHYTOCHROME-INTERACTING FACTOR 3/4/7 (PIF3/4/7), ETHYLENE-INSENSITIVE 3 (EIN3), and MYB15 [13]. MYB15 interacts with ICE1 and directly represses *CBF* gene expression by directly binding to their promoters [13]. The U-box E3 ligases PUB25 and PUB26 promote LT stress tolerance by targeting MYB15 for degradation under cold stress [13]. Phytohormone signaling pathways also play an important role in LT stress responses in plants [14]. For example, brassinolides (BRs) protect plants from LT stress by improving photosynthesis [15]. BR improves LT stress tolerance by modulating ABA contents through increases in the expression of *NCED1* in tomato [16]. Furthermore, BIN2 mediates the crosstalk between BR and ABA in response to LT stress in plants [16]. Ethylene signaling negatively regulates LT stress tolerance by EIN3-mediated repression of *CBF* expression and type-A *ARR* genes, which are negative regulators of cytokinin signaling. The F-box subunits of the SCF complex directly target EIN3 for degradation under LT stress, thus modulating freezing tolerance [7,17,18].

Humic acids, known as plant growth stimulants, are a class of macromolecular substances widely found in the natural environment, formed by plant and animal residues through complex biochemical processes, with complex structures and a variety of active functional groups, and thus have a strong ion exchange and adsorption ability [19]. Many studies have demonstrated that humic acid affects root growth and nutrient uptake in roots [19,20,21,22] and modulates plant growth and development via the auxin pathway [23]. Humic acids improve stress tolerance in plants by reducing reactive oxygen species (ROS) overaccumulation and malondialdehyde (MDA) contents, thereby reducing plasma membrane permeability [19,21]. Supplementation with humic acids increased chlorophyll contents and improved thylakoid structure, thus promoting photosynthetic efficiency [20]. Humic acids promoted plant growth and nutrition assimilation by inducing the expression of the *H^+^-ATPase* gene and genes encoding enzymes involved in nitrogen conversion, transport, and organic acid synthesis, such as *asparagine synthetase* and *phenylalanine deaminase* in maize [21,22]. In the presence of low molecular weight humic acid, the expression of genes related to nitrate absorption (*NRT2.1* and *MHA2*) and nitrate assimilation genes (*NR1*) was upregulated in maize roots [24]. Humic acid application also improved drought stress tolerance by inducing *OsTIP* expression in rice [21].

Zucchini (*Cucurbita pepo* L.) is one of the main vegetable crops cultivated in facilities in northern China. LT stress reduces the yield and quality of zucchini by inhibiting the photosynthetic rate, accelerating chlorophyll decomposition, and inducing oxidative damage to the cell membrane in zucchini seedlings [9,25,26,27]. In the breeding and cultivation of zucchini, it is important to find suitable germplasm resources or use reasonable fertilization or cultivation modes to alleviate the adverse effects caused by LT stress [28,29]. In this study, we investigated the physiological and molecular mechanisms underlying humic acid-mediated LT stress tolerance in zucchini seedlings.

## 2. Results and Discussion

### 2.1. Humic Acids Alleviate Growth Inhibition of Zucchini Seedlings under LT Stress

**Figure 1:**
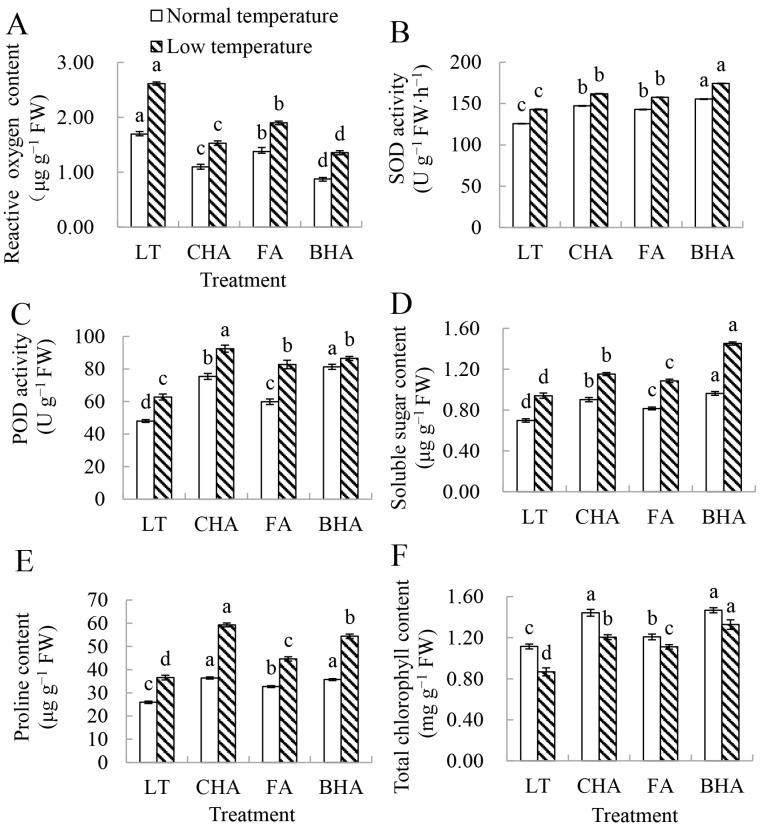
Effects of humic acid treatment on the (**A)** reactive oxygen contents, (**B**) SOD activity, (**C**) POD activity, (**D**) soluble sugar contents, (**E**) proline contents, and (**F**) total chlorophyll contents in zucchini leaves under LT stress (*p* < 0.05). LT, 5 °C. CHA, 5 °C + 0.05% coal-based humic acid. FA, 5 °C+ 0.05% fulvic acid. BHA, 5 °C + 0.05% biochemical humic acid. Error bars represented the ± SEs (*p* < 0.05). Different letters indicate significantly different values at *p* < 0.05, according to Tukey’s test.

We first investigated the physiological indexes of zucchini seedlings under LT with or without humic acid treatments. LT stress-induced, while supplementation with three kinds of different humic acids markedly reduced the accumulation of reactive oxygen species in the leaves (Figure 1A). We then examined the changes in the antioxidative enzyme activities in zucchini leaves. LT stress markedly induced the activities of superoxide dismutase (SOD) and catalase (CAT); supplementation with CHA, FA, and BHA further increased their activities in zucchini seedlings under LT stress (Figure 1B,C). SOD activities in the leaves of CHA-, FA-, and BHA-treated plants were 13.2%, 10.2%, and 22.1% higher than those in LT-treated plants (LT), and peroxidase (POD) activities in the leaves of CHA-, FD-, and BHA-treated plants were 38.1%, 32.3%, and 48.2% higher than those in LT, respectively (Figure 1B,C). SOD catalyzes the conversion of superoxide free radicals into H_2_O_2_, and then finally converts H_2_O_2_ into H_2_O through CAT and POD [9,10]. The increased activities of SOD, CAT, and POD could decrease the reactive oxygen species (ROS) levels in leaves, thereby improving LT stress tolerance in zucchini seedlings.

Soluble sugar and proline are two important osmotic protective substances in plant cells [30,31]. The soluble sugar contents in the leaves of CHA-, FA-, and BHA-treated plants were 22%, 15%, and 54% higher than those in LT, respectively (Figure 1D). The proline content in the leaves of CHA-, FA-, and BHA-treated plants was 63.3%, 22.5%, and 50.2% higher than that in LT, respectively (Figure 1E). These results indicated that supplementation with different kinds of humic acids increased the contents of osmotic protective substances; thus, it will be beneficial to improve the tolerance of seedlings to LT stress in zucchini seedlings.

In addition, LT stress also markedly reduces the chlorophyll content of zucchini leaves. Supplementation with CHA, FA, and BHA increased chlorophyll contents by 50.2%, 37.3%, and 62.1% compared with those in chilling-treated CKD, respectively (Figure 1F). Taken together, these results indicated that humic acids alleviate LT-induced oxidative damage by increasing the activities of antioxidant enzymes and the contents of soluble sugars and proline in zucchini leaves.

### 2.2. Transcriptomic Analysis

**Figure 2:**
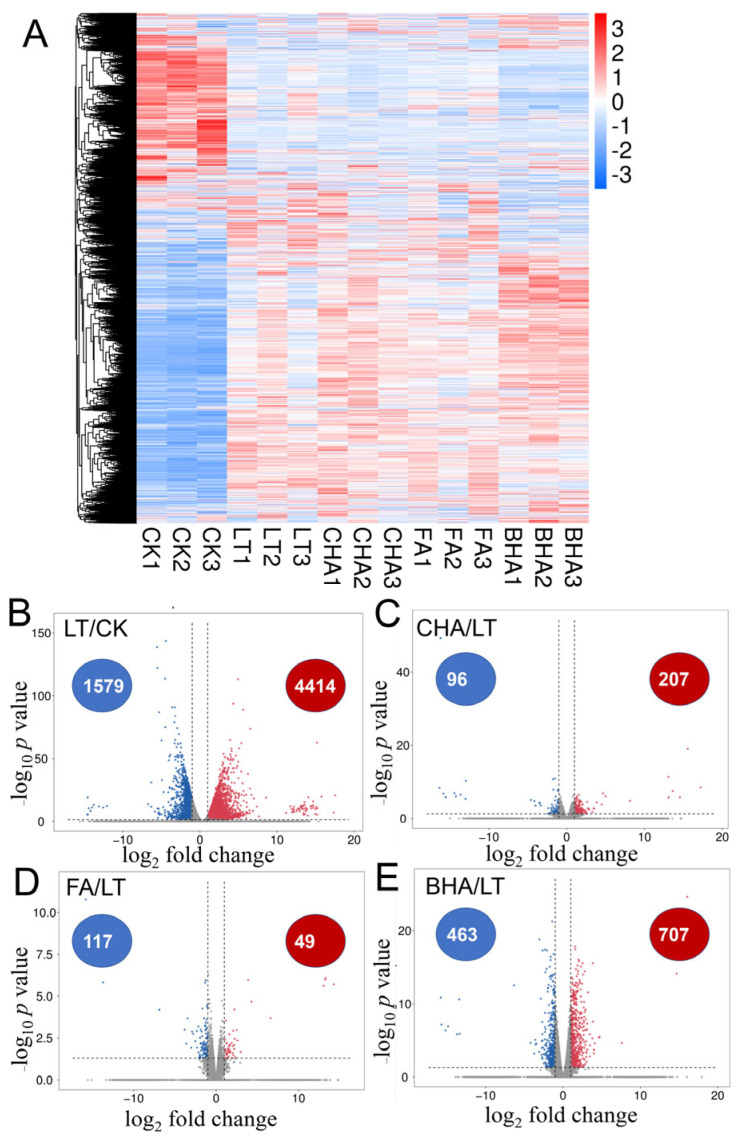
Transcriptomic analysis to determine the differentially expressed genes (DEGs) in the leaves of coal-based humic acid (CHA)-, fulvic acid (FA)-, and biochemical humic acid (BHA)-treated zucchini seedlings under LT stress. (**A**) Hierarchical clustering analysis of the transcriptome data. (**B**–**E**) Volcano map of DEGs. The scale represents log_10_ (FPKM+1). (**B**) LT/CK comparison. (**C**) CHA/LT comparison. (**D**) FA/LT comparison. (**E**) BHA/LT comparison. The log_2_-fold change (FC) ≥ 1 or ≤ −1 and FDR < 0.05. LT, 5 °C. CHA, 5 °C + 0.05% coal-based humic acid. FA, 5 °C + 0.05% fulvic acid. BHA, 5 °C + 0.05% biochemical humic acid.

To better elucidate the molecular mechanisms underlying humic acid-alleviated oxidative damage caused by LT stress, a transcriptomic analysis was performed to detect the differentially expressed genes (DEGs) in zucchini leaves (Figure 2A). The original sequencing data of the 15 processed sequencing samples ranged from 7.22 to 8.30 GB, and the effective data after filtering the original data ranged from 6.53 to 7.59 GB. The sequencing quality analysis showed that the detection rate of Q30 was > 98% and the detection rate of an effective read was > 80%, which met the requirements of expression pattern analysis. The density of gene transcript expression values of different zucchini samples was in line with a normal distribution, and the gene expression trend of biologically duplicate zucchini samples tended to be consistent (Figure S1); the correlation analysis showed the differential expression pattern of genes in humic acid-treated plants (Figure S2). 

After bioinformatics analysis, a total of 27,868 transcripts were collected, of which 25,500 and 9,123 transcripts were annotated from the GO and KEGG databases, respectively. Compared with the untreated control, a total of 5993 DEGs (including 4414 upregulated and 1759 downregulated) were identified in the CKD treatment. Compared with the CKD treatment, a total of 166 DEGs (including 49 upregulated and 117 downregulated), 303 DEGs (including 207 upregulated and 96 downregulated), and 1170 DEGs (including 707 upregulated and 463 downregulated) were identified in the FD, HD, and BD treatments, respectively (Figure 2B–E; Tables S2–S5).

### 2.3. Humic Acids Modulate Metabolism, Stress Responses and Phytohormone Pathways under LT Stress

**Figure 3:**
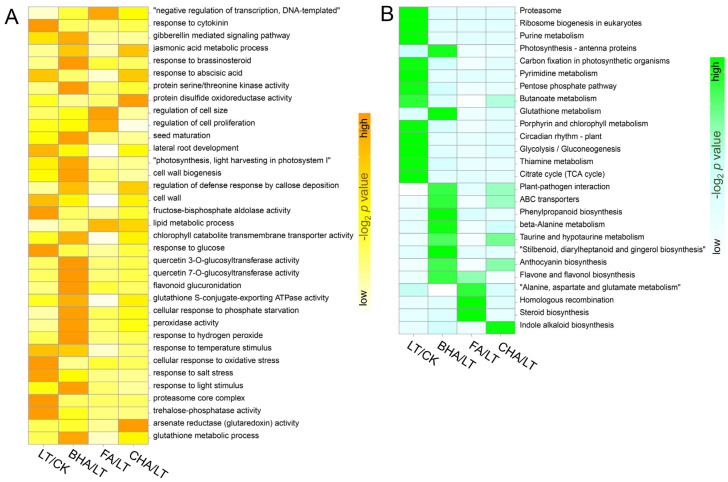
GO (**A**) and KEGG (**B**) pathway analyses. LT, 5 °C. CHA, 5 °C + 0.05% coal-based humic acid. FA, 5 °C+ 0.05% fulvic acid. BHA, 5 °C + 0.05% biochemical humic acid.

Gene ontology (GO) analysis showed that DEGs related to transcriptional regulation, phytohormone signaling pathways, antioxidative regulation, and flavonoid metabolism were highly enriched in the ‘BHA/LT’ comparison (Figure 3A). The DEGs related to the regulation of the RNA polymerase II transcription complex, protein refolding, proteasome assembly, ATPase activity, and threonine-type endopeptidase activity were enriched in the ‘LT/CK’ comparison (Figure S3); DEGs related to glutamic acid and jasmonic acid metabolic processes and stress responses were enriched in the ‘CHA/LT’ comparison (Figure S4); DEGs related to cell growth and proliferation, cell wall, and transcriptional regulation were enriched in the ‘FA/LT’ comparison (Figure S5); and DEGs related to the plasma membrane, protein serine/threonine kinase activity, ATPase activity, and stress responses were enriched in the ‘BHA/LT’ comparison (Figure S6).

The Kyoto Encyclopedia of Genes and Genomes (KEGG) pathway analysis showed that DEGs associated with photosynthesis, glutathione metabolism, phenylpropanoid biosynthesis, and flavonoid metabolism pathways were enriched in the ‘LT/CK’ comparison (Figure 3B; Figure S7); DEGs associated with plant-pathogen interaction, MAPK signaling pathway, glutathione metabolism, and anthocyanin biosynthesis were enriched in the ‘CHA/LT’ comparison (Figure 3B; Figure S8); DEGs associated with amino acid metabolism and sugar metabolism were enriched in the ‘FA/LT’ comparison (Figure 3B; Figure S9); and DEGs associated with photosynthesis, glutathione metabolism, and phenylpropanoid biosynthesis were enriched in the ‘BHA/LT’ comparison (Figure 3B; Figure S10). Taken together, these results indicated that humic acids improve LT stress tolerance by reprogramming metabolism and modulating stress responses and signal transduction pathways in zucchini leaves.

**Figure 4:**
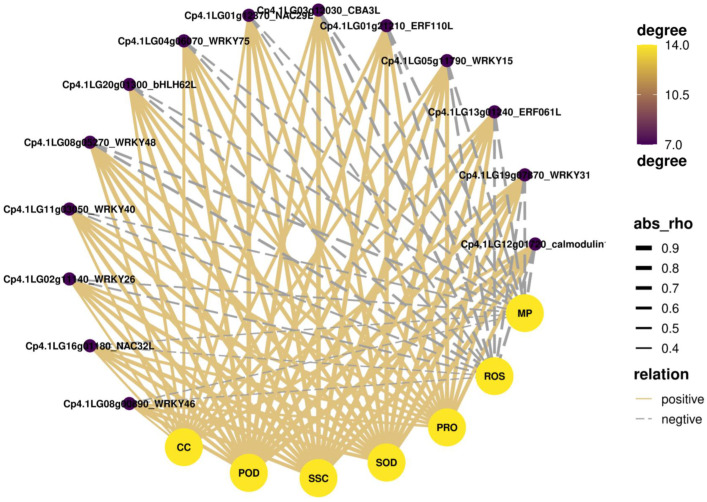
Correlation analysis between the 14 common differentially expressed TFs in FA/LT, CHA/LT, and BHA/LT comparisons and the physiological indexes. CC, chlorophyll content. POD, peroxidase. SSC, soluble sugar contents. SOD, superoxide dismutase. PRO, proline. ROS, reactive oxygen species. MP, plasma membrane permeability.

Comparative analysis showed that the three kinds of humic acids have some similar biological functions in modulating the LT stress response in zucchini, while BHA had the strongest effect compared to FA and CHA (Figure 3). Transcriptomic analysis showed that seventeen DEGs were coexpressed in FA/LT, CHA/LT, and BHA/LT comparisons, including the genes involved in calmodulin signaling, two ethylene-responsive transcription factors (TFs), two peroxidase genes, and ten TFs, including two *NAC* genes (*Cp4.1LG01712370* and *Cp4.1LG16g01180*), seven *WRKY* genes (*Cp4.1LG05g11790*, *Cp4.1LG02g11140*, *Cp4.1LG19g07870*, *Cp4.1LG11g03050*, *Cp4.1LG08g00890*, *Cp4.1LG08270,* and *Cp4.1LG04g06070*) and an *MYB* gene (*Cp4.1LG20g01300*) (Table S6). Phytohormones and calmodulin signaling play an important role in the response to LT stress in plants [32]. Previous studies have shown that ethylene regulates cold tolerance in tomatoes [17,18], and overexpression of the *TERF2/LeERF2* gene reduces freezing damage in tobacco and tomatoes [18]. In this study, we found that the expression of two ethylene-responsive TF genes (*Cp4.1LG13g01240* and *Cp4.1LG01g21210*), a calmodulin gene (*Cp4.1LG12g01720*), and two calmodulin-binding transcription activator genes (*Cp4.1LG15g02270* and *Cp4.1LG03g12030*) were induced in the FA/LT, CHA/LT, and BHA/LT comparisons (Table S6), indicating that treatment with FA, CHA, and BHA improved LT stress tolerance by regulating the ethylene signaling pathway and the calmodulin signal transduction pathway under LT stress. In addition, the expression of genes related to glutathione metabolism, peroxidase synthesis, and other antioxidant accumulation was upregulated in the FA/LT, CHA/LT, and BHA/LT comparisons, suggesting that humic acids improved the antioxidant capacity and further enhanced LT tolerance, which supported the phenotypic and physiological analysis results (Figure 1). To further elucidate the potential correlation between the differentially expressed genes and physiological changes in humic acid-treated plants, we next performed a correlation analysis between the 14 common differentially expressed TFs and the physiological indexes, including antioxidative enzyme activities, osmotic regulators, ROS, soluble sugar, chlorophyll contents, and the chilling damage index. All TFs showed a positive correlation with antioxidative enzyme activities, osmotic regulators, soluble sugar, and chlorophyll contents; in contrast, they showed a negative correlation with ROS and the chilling damage index (Figure 4). These results collectively suggested that these TFs play important roles in modulating LT tolerance by regulating antioxidative capacity and reprogramming plant metabolism in zucchini seedlings.

### 2.4. Weighted Co-Expression Network Analysis Identified Hub Genes Associated with Humic AC-Id-Mediated ROS Scavenging and Osmotic Protection 

**Figure 5:**
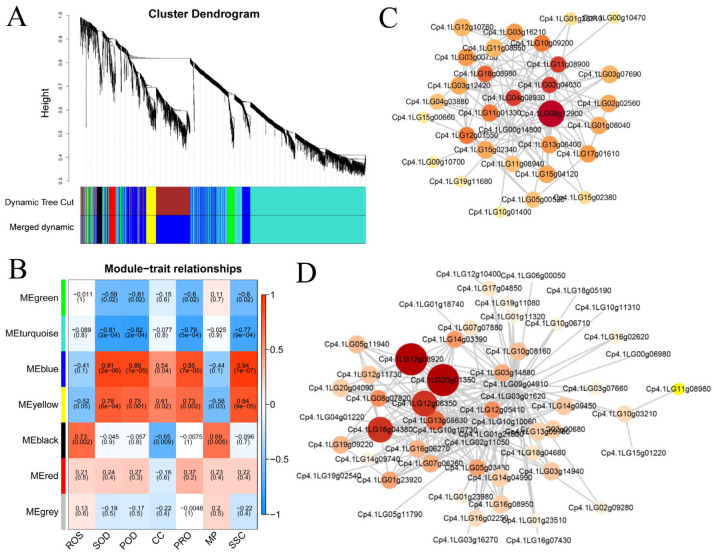
Weighted gene coexpression network analysis of DEGs in zucchini leaves. (**A**) Hierarchical clustering tree and heatmap of genes were constructed by correlation coefficients between genes. Different branches of the clustering tree represent different gene modules, and different colors represent different modules. (**B**) Correlation between the physio-biochemical indexes and each module. Module names are displayed on the Y-axis, and the physio-biochemical indexes are displayed on the X-axis. The depth of the color indicates the degree of correlation (red, positive correlation; blue, negative correlation). CC, chlorophyll content. PRO, proline. MP, membrane-lipid peroxidation damage. SSC, soluble sugar contents. (**C,D**) The circle diagram shows the predicted regulatory network for the yellow module (**C**) and blue module (**D**).

Weighted gene coexpression network analysis (WGCNA) was performed on the DEGs to mine the core genes (Hub genes) (Figure 5A; Figure S11); subsequently, the correlation between the modules and the physio-biochemical indexes was analyzed (Figure 5B). A soft threshold power of 6 was introduced in the network topology to reveal the scale independence and average connectivity of the network (Figure S10). Hierarchical clustering trees were constructed using weighted correlation coefficients between genes (Figure 5A). DEGs from the 15 samples were clustered and divided into seven modules (Figure 5B). Analysis of the correlations of the seven modules and seven physiological indicators showed that the MEblue module and the MEyellow module were highly positively correlated with SOD, POD, proline, and soluble sugar, whereas they showed a negative correlation with ROS and membrane-lipid peroxidation damage. 

In contrast, the Meturquoise module and the Megreen module showed negative correlations with SOD, POD, proline, and soluble sugars. The Meblack module had a high positive correlation with ROS, while it showed a moderate negative correlation with chlorophyll content, indicating that genes in this module play a major role in modulating ROS levels. The poor correlation of the Mered module and the Megray module with these substances suggests that the DEGs in the two modules might not be involved in the regulation of these substances. Based on the above analysis, a coexpression network was further constructed for the Meblue and Meyellow modules (Figure 5C,D). Based on the coexpression network, *Cp4.1LG12g08920* (*beta-fructofuranosidase*), *Cp4.1LG20g01350*, *Cp4.1LG08g12900*, *Cp4.1LG16g04380*, *Cp4.1LG12g06350*, *Cp4.1LG04g08930* (*chitotriosidase-1-like*), *Cp4.1LG02g04030,* and *Cp4.1LG11g08900* (*F-box protein PP2-B15-like*) genes were screened as potential Hub genes. The beta-fructofuranosidase, also called invertase, catalyzes the degradation of sucrose into fructose and glucose and participates in osmotic regulation and the accumulation of sugar in cells [33]; thus, increased expression of the *beta-fructofuranosidase* gene improves the osmotic adjustment ability of leaves under LT stress. These results collectively indicated that these genes played a key role in the response to LT stress. Future studies will elucidate the function of these genes and their roles in modulating humic acid-mediated LT tolerance in zucchini.

## 3. Materials and Methods

### 3.1. Plant Materials and Experimental Treatment

Seeds of the zucchini (*Cucurbita pepo* L.) cultivar *SH-3* were germinated and grown in 1/4 Hoagland solution in Petri dishes. Coal-based humic acid (CHA) was purchased from Luliang Shengda Biological Co. Ltd. (including H_2_O = 14.96%, black humic acid = 51.98%, brown humic acid = 2.33%, yellow humic acid = 0.69%, and K_2_O = 11.8%). Biochemical humic acid (BHA) was purchased from Fujian Oasis Co. Ltd. (including H_2_O = 35.21%, fulvic acid = 32.64%, and K_2_O = 8.92%). Fulvic acid (FA) was purchased from Shanxi Linhai Co. Ltd. (including H_2_O = 9.24%, brown fulvic acid = 3.61%, fulvic acid = 47.22%, and K_2_O = 12.53%). After seed emergence, twelve-d-old seedlings at identical growth stages (two leaves and one heart) were chosen for the following experiments. The experiment was divided into five treatment groups: (1) 20 °C (CK); (2) 5 °C (LT); (3) 5 °C + 0.05% CHA; (4) 5 °C+ 0.05% FA; and (5) 5 °C + 0.05% BHA. The light intensity was 400 uml.m^−2^.s^−1^, and the light-dark cycle was 12 h/12 h. After three days of treatment in 1/4 Hoagland solution, the leaves were collected for physiological and transcriptomic analysis.

### 3.2. Determination of the Degree of Oxidative Damage, Antioxidative Enzyme Activities, and Soluble Sugar, Proline and Chlorophyll Contents

The oxidative damage degree was evaluated using the conductance method [34]. Superoxide dismutase (SOD) activity was determined using the nitroblue tetrazolium reduction method [35]. Briefly, approximately 0.3 g of fresh leaves was ground in 1.5 mL of 62.5% phosphoric acid buffer (pH 7.8) in an ice bath and centrifuged for 15 min at 15,000 r/min, and then the supernatant was used for SOD activity determination. Approximately 0.3 g of fresh tissue was ground in 8 mL of 0.05 M sodium phosphate buffer (pH 7.8) and centrifuged at 10,000× g for 15 min; the supernatant was used as the crude enzyme extract for peroxidase (POD) activity determination as described by Guan et al., (2009) [36]. The reaction mixture contained 2.7 mL of 25 mM sodium phosphate buffer (pH 7.0, containing 2 mM EDTANa_2_), 100 μL of 1.5% guaiacol, 100 μL of 300 mM H_2_O_2_, and 100 μL enzyme extract [36]. Soluble sugar contents were determined using the anthrone colorimetric method [37]. For proline content determination, approximately 0.3 g of leaf tissue was homogenized with liquid nitrogen, and the tissue powder was suspended in 1 mL of 3% sulfosalicylic acid. After centrifugation at 4 °C at 1000× *g* for 5 min, 0.1 mL of the supernatant was mixed with 0.2 mL of acidic ninhydrin, 0.2 mL of 96% acetic acid, and 0.1 mL of 3% sulfosalicylic acid. The mixture was incubated at 96 °C for 1 h, mixed with 1 mL toluene, and centrifuged at 4 °C at 1000× *g* for 5 min. The upper phase was collected, and the absorbance was read at 520 nm. Then, the proline content was calculated as described by Tiwari et al., (2020) [38]. Chlorophyll contents were determined using the ethanol extraction method [39].

### 3.3. Transcriptomic Analysis

Total RNA was extracted from the leaves of zucchini seedlings using the TRIzol method (Takara) (Table S1). mRNA was enriched by magnetic beads linked with Oligo (DT) after the total RNA was qualified. The extracted mRNA was randomly broken into short fragments by a fragment buffer. The fragmented mRNA was used as a template to synthesize a single strand of cDNA with primers containing six random bases, followed by the addition of buffer, dNTPs, RNaseH, and DNA polymerase I to synthesize the second strand of cDNA. The double-stranded products were purified with ampure XP magnetic beads. The sticky end of DNA was repaired to the flat end by T4 DNA polymerase and Klenow DNA polymerase activity. Base A was added to the 3’ end, and the adaptor was added. Ampure XP magnetic beads were used for fragment selection, and PCR amplification was performed to obtain the final sequencing library. Illumina NovaseqTM6000 was used for sequencing after the library was qualified. Transcript assembly and differential gene expression analysis were performed using the Cufflinks software package (version 2.2.1, Cole Trapnell lab, University of Washington). The raw data were archived at the Short Read Archive of the National Center for Biotechnology Information under accession no. PRJNA906041. Transcripts were assembled based on comparison with the reference genome of zucchini (ftp://cucurbitgenomics.org/pub/cucurbit/genome/Cucurbita_pepo/) and using HISAT2 2.0.5 (https://ccb.jhu.edu/software/hisat2, accessed on 10 September 2022 [40]). The RNA-seq sequencing result is the sequence fragments (Reads) of the expressed transcripts. Generally, the more transcripts a gene expresses, the more Reads from the gene will be obtained. Therefore, the expression levels of the genes can be calculated according to the number (Count) of the sequenced reads compared to each transcript. However, the number of gene read counts is also related to gene length and sequencing depth. To ensure that the number of fragments truly reflected the transcript expression level, it was necessary to normalize the number of Mapped Reads and the transcript length in the sample. The expression levels were estimated by the StringTie program (https://ccb.jhu.edu/software/hisat2, accessed on 10 September 2022 [41]) and FPKM (Fragments Per Kilobase of script per Million fragments mapped) for standardization as an indicator to measure the level of transcripts or gene expression [42], and the differentially expressed genes (DEGs) were analyzed based on the Count value of genes in each sample [43]. DESeq2 software was used for differential analysis. Fold Change ≥ 2 and FDR < 0.05 are used as screening criteria. Gene Ontology (GO) analysis (http://geneontology.org, accessed on 10 September 2022) and Kyoto Encyclopedia of Genes and Genomes (KEGG) (http://www.kegg.jp/kegg, accessed on 10 September 2022) enrichment analysis were performed to identify the biological function and pathways. The Pearson Correlation Analysis was completed by using the online software at https://www.omicshare.com/tools/ (accessed on 10 September 2022).

### 3.4. Statistics and Analysis

The experiment was repeated three times, with 14 seedlings for each repetition. The Microsoft Office Excel 2016 package (Redmond, WA, USA) was used to analyze the data. A one-way ANOVA with Tukey’s test was conducted to determine the significance (*p* < 0.05) using SPSS 25.0. The data are presented as the mean (± SE) of at least three biological replications. For the transcriptomic analysis, the thresh old was set to a log_2_ fold change (FC) ≥ 1 or ≤ −1 and FDR < 0.05.

## 4. Conclusions

In summary, supplementation with humic acids increased antioxidative enzyme activities and the contents of soluble sugar and proline, thereby decreasing the degree of oxidative damage in leaves and increasing chlorophyll contents, finally improving plant growth under LT stress in zucchini seedlings. Meanwhile, humic acids improved LT tolerance through the modulation of calmodulin signaling and phytohormone signaling in plants. Several TFs, including two *NAC* genes, seven *WRKY* genes, and an *MYB* gene, were identified as the candidate key genes involved in humic acid-mediated LT stress tolerance in zucchini seedlings. Moreover, in support of the physiological results, humic acids also induced the expression of genes related to glutathione metabolism, peroxidase synthesis, and other antioxidant accumulation, thereby decreasing ROS accumulation in plants. Humic acids consist of a variety of active functional groups, and the relationship between their structure and function, how humic acids modulate LT tolerance in zucchini seedlings, and the molecular mechanisms underlying these Hub gene-mediated responses to LT stress still need to be further elucidated.

## Data Availability

The data is contained within the manuscript and Supplementary Materials.

## Supplementary Materials

The following supporting information can be downloaded at: https://www.mdpi.com/article/10.3390/plants12030548/s1, Figure S1: Expression density of transcripts in zucchini samples. Figure S2: GO enrichment of DEGs in LT/CK comparison. Figure S3: GO enrichment of DEGs in CHA/LT comparison. Figure S4: GO enrichment of DEGs in FA/LT comparison. Figure S5: GO enrichment of DEGs in BHA/LT comparison. Figure S6: KEGG pathway of DEGs in LT/CK comparison. Figure S7: KEGG pathway of DEGs in CHA/LT comparison. Figure S8: KEGG pathway of DEGs in FA/LT comparison. Figure S9: KEGG pathway of DEGs in BHA/LT comparison. Figure S10: The soft threshold with scale independence and mean connectivity. Table S1: List of RNA-sequencing analysis libraries. Table S2: Gene expression in LT/CK comparison. Table S3: Gene expression in CHA/LT comparison. Table S4: Gene expression in FA/LT comparison. Table S5: Gene expression in BHA/LT comparison. Table S6: Common DEGs in FA/LT, CHA/LT, and BHA/LT comparisons.

## References

[1] Chen Y., Zhang M., Chen T., Zhang Y., An L. (2006). The Relationship between Seasonal Changes in Anti-Oxidative System and Freezing Tolerance in the Leaves of Evergreen Woody Plants of Sabina. South Afr. J. Bot..

[2] Ding Y., Yang S. (2022). Surviving and Thriving: How Plants Perceive and Respond to Temperature Stress. Dev. Cell.

[3] Partelli F.L., Batista-Santos P., Scotti-Campos P., Pais I.P., Quartin V.L., Vieira H.D., Ramalho J.C. (2011). Characterization of the Main Lipid Components of Chloroplast Membranes and Cold Induced Changes in *Coffea* spp.. Environ. Exp. Bot..

[4] Gu Y., He L., Zhao C., Wang F., Yan B., Gao Y., Li Z., Yang K., Xu J. (2017). Biochemical and Transcriptional Regulation of Membrane Lipid Metabolism in Maize Leaves under Low Temperature. Front. Plant Sci..

[5] Chinnusamy V., Ohta M., Kanrar S., Lee B.-H., Hong X., Agarwal M., Zhu J.-K. (2003). ICE1: A Regulator of Cold-Induced Transcriptome and Freezing Tolerance in Arabidopsis. Genes Dev..

[6] Jeon J., Kim N.Y., Kim S., Kang N.Y., Novák O., Ku S.-J., Cho C., Lee D.J., Lee E.-J., Strnad M. (2010). A Subset of Cytokinin Two-Component Signaling System Plays a Role in Cold Temperature Stress Response in Arabidopsis. J. Biol. Chem..

[7] Shi Y., Tian S., Hou L., Huang X., Zhang X., Guo H., Yang S. (2012). Ethylene Signaling Negatively Regulates Freezing Tolerance by Repressing Expression of CBF and Type-A ARR Genes in Arabidopsis. Plant Cell..

[8] Hincha D.K., Zuther E. (2020). Introduction: Plant Cold Acclimation and Winter Survival. Methods Mol. Biol..

[9] Shan C., Zhang S., Ou X. (2018). The Roles of H_2_S and H_2_O_2_ in Regulating AsA-GSH Cycle in the Leaves of Wheat Seedlings under Drought Stress. Protoplasma.

[10] Neill S.J. (2002). Hydrogen Peroxide and Nitric Oxide as Signalling Molecules in Plants. J. Exp. Bot..

[11] Bajwa V.S., Shukla M.R., Sherif S.M., Murch S.J., Saxena P.K. (2014). Role of Melatonin in Alleviating Cold Stress in Arabidopsis Thaliana. J. Pineal. Res..

[12] Pennycooke J.C., Cheng H., Roberts S.M., Yang Q., Rhee S.Y., Stockinger E.J. (2008). The Low Temperature-Responsive, Solanum CBF1 Genes Maintain High Identity in Their Upstream Regions in a Genomic Environment Undergoing Gene Duplications, Deletions, and Rearrangements. Plant Mol. Biol..

[13] Zhang L., Jiang X., Liu Q., Ahammed G.J., Lin R., Wang L., Shao S., Yu J., Zhou Y. (2020). The HY5 and MYB15 Transcription Factors Positively Regulate Cold Tolerance in Tomato via the CBF Pathway. Plant Cell Environ..

[14] Eremina M., Rozhon W., Poppenberger B. (2016). Hormonal Control of Cold Stress Responses in Plants. Cell Mol. Life Sci..

[15] Fang P., Yan M., Chi C., Wang M., Zhou Y., Zhou J., Shi K., Xia X., Foyer C.H., Yu J. (2019). Brassinosteroids Act as a Positive Regulator of Photoprotection in Response to Chilling Stress. Plant Physiol..

[16] An S., Liu Y., Sang K., Wang T., Yu J., Zhou Y., Xia X. (2022). Brassinosteroid Signaling Positively Regulates Abscisic Acid Biosynthesis in Response to Chilling Stress in Tomato. J. Integr. Plant Biol..

[17] Joseph A.C., Deikman J., Michael D. (1997). Orzolek. Increased ethylene synthesis enhances chilling tolerance in tomato. Physiol. Plant..

[18] Zhang Z.J., Huang R.F. (2010). Enhanced Tolerance to Freezing in Tobacco and Tomato Overexpressing Transcription Factor TERF2/LeERF2 Is Modulated by Ethylene Biosynthesis. Plant Mol. Biol..

[19] Muscolo A., Sidari M., Nardi S. (2013). Humic Substance: Relationship between Structure and Activity. Deeper Information Suggests Univocal Findings. J. Geochem. Explor..

[20] Piccolo A., Nardi S., Concheri G. (1992). Structural Characteristics of Humic Substances as Related to Nitrate Uptake and Growth Regulation in Plant Systems. Soil Biol. Biochem..

[21] García A.C., Santos L.A., Izquierdo F.G., Sperandio M.V.L., Castro R.N., Berbara R.L.L. (2012). Vermicompost Humic Acids as an Ecological Pathway to Protect Rice Plant against Oxidative Stress. Ecol. Eng..

[22] Vaccaro S., Ertani A., Nebbioso A., Muscolo A., Quaggiotti S., Piccolo A., Nardi S. (2015). Humic Substances Stimulate Maize Nitrogen Assimilation and Amino Acid Metabolism at Physiological and Molecular Level. Chem. Biol. Technol. Agric..

[23] Trevisan S., Francioso O., Quaggiotti S., Nardi S. (2010). Humic Substances Biological Activity at the Plant-Soil Interface: From Environmental Aspects to Molecular Factors. Plant Signal Behav..

[24] Quaggiotti S., Ruperti B., Pizzeghello D., Francioso O., Tugnoli V., Nardi S. (2004). Effect of Low Molecular Size Humic Substances on Nitrate Uptake and Expression of Genes Involved in Nitrate Transport in Maize (*Zea mays* L.). J. Exp. Bot..

[25] Miquel M., James D., Dooner H., Browse J. (1993). Arabidopsis Requires Polyunsaturated Lipids for Low-Temperature Survival. Proc. Natl. Acad. Sci. USA.

[26] Chen M., Thelen J.J. (2013). ACYL-LIPID DESATURASE2 Is Required for Chilling and Freezing Tolerance in Arabidopsis. Plant Cell.

[27] Liu J., Wang B., Li Y., Huang L., Wen Q. (2020). RNA Sequencing Analysis of Low Temperature and Low Light Intensity-Responsive Transcriptomes of Zucchini (*Cucurbita pepo* L.). Sci. Hortic..

[28] Palma F., Carvajal F., Jamilena M., Garrido D. (2016). Putrescine Treatment Increases the Antioxidant Response and Carbohydrate Content in Zucchini Fruit Stored at Low Temperature. Postharvest Biol. Technol..

[29] Sánchez-Bel P., Egea I., Sánchez-Ballesta M.T., Martinez-Madrid C., Fernandez-Garcia N., Romojaro F., Olmos E., Estrella E., Bolarín M.C., Flores F.B. (2012). Understanding the Mechanisms of Chilling Injury in Bell Pepper Fruits Using the Proteomic Approach. J. Proteom..

[30] Rosa M., Prado C., Podazza G., Interdonato R., González J.A., Hilal M., Prado F.E. (2009). Soluble Sugars--Metabolism, Sensing and Abiotic Stress: A Complex Network in the Life of Plants. Plant Signal Behav..

[31] Boriboonkaset T., Theerawitaya C., Yamada N., Pichakwm A., Supaibulwatana K., Cha-um S., Takabe T., Kirdmanee C. (2013). Regulation of some carbohydrate metabolism-related genes, starch and soluble sugar contents, photosynthetic activities and yield attributes of two contrasting rice genotypes subjected to salt stress. Protoplasma.

[32] Rambod A., Noor A.S., Mahmood M., Zetty N.B.Y., Narges A., Mahbod S., Alireza V., Nahid K., Mohamed M.H. (2017). Role of ethylene and the APETALA 2/ethylene response factor superfamily in rice under various abiotic and biotic stress conditions. Environ. Exp. Bot..

[33] Xu Z.S., Chen X.J., Lu X.P., Zhao B.P., Yang Y.M., Liu J.H. (2021). Integrative analysis of transcriptome and metabolome reveal mechanism of tolerance to salt stress in oat (*Avena sativa* L.). Plant Physiol. Biochem..

[34] Melillo M.T., Leonetti P., Bongiovanni M., Castagnone-Sereno P., Bleve-Zacheo T. (2006). Modulation of Reactive Oxygen Species Activities and H _2_ O _2_ Accumulation during Compatible and Incompatible Tomato–Root-knot Nematode Interactions. New Phytol..

[35] Sepehri E., Hosseini B., Hedayati A. (2022). The Effect of Iron Oxide Nano-Particles on the Production of Tropane Alkaloids, H6h Gene Expression and Antioxidant Enzyme Activity in Atropa Belladonna Hairy Roots. Russ. J. Plant Physiol..

[36] Guan Y., Hu J., Wang X., Shao C. (2009). Seed Priming with Chitosan Improves Maize Germination and Seedling Growth in Relation to Physiological Changes under Low Temperature Stress. J. Zhejiang Univ. Sci. B.

[37] Pedro G.C., Alfonso L., Elodie H., María J.L., María Teresa L. (2020). Effects of Exogenous Application of Osmotic Adjustment Substances on Growth, Pigment Concentration, and Physiological Parameters of Dracaena Sanderiana Sander under Different Levels of Salinity. Agronomy.

[38] Tiwari A., Rastogi A., Singh V., Arunachalam A. (2020). Effect of Water Stress on Oxidative Damage and Antioxidant Enzyme Activity in Finger Millet and Barnyard Millet. Indian J. Hill Farming.

[39] Kaya C., Akram N.A., Ashraf M., Sonmez O. (2018). Exogenous Application of Humic Acid Mitigates Salinity Stress in Maize (*Zea mays* L.) Plants by Improving Some Key Physico-Biochemical Attributes. Cereal Res. Commun..

[40] Kim D., Langmead B., Salzberg S.L. (2015). HISAT: A Fast Spliced Aligner with Low Memory Requirements. Nat. Methods..

[41] Pertea M., Pertea G.M., Antonescu C.M., Chang T.C., Mendell J.T., Salzberg S.L. (2015). StringTie Enables Improved Reconstruction of a Transcriptome from RNA-Seq Reads. Nat. Biotechnol..

[42] Trapnell C., Williams B.A., Pertea G., Mortazavi A., Kwan G., Van Baren M.J., Salzberg S.L., Wold B.J., Pachter L. (2010). Transcript assembly and quantification by RNA Seq reveals unannotated transcripts and isoform switching during cell differentiation. Nat. Biotechnol..

[43] Love M.I., Huber W., Anders S. (2014). Moderated estimation of fold change and dispersion for RNA-seq data with DESeq2. Genome Biol..

