# Supplementary material for: Physiological and Transcriptomic Analyses Revealed That Humic Acids Improve Low-Temperature Stress Tolerance in Zucchini (Cucurbita pepo L.) Seedlings
Source: Plants (Basel). 2023 Jan 25;12(3):548. doi: 10.3390/plants12030548 (PMC9921430; doi:10.3390/plants12030548)
Supplement: Supplementary file 1 [file plants-12-00548-s001.zip › plants-2090779 Supplementary Figures.pdf]

Supplementary Materials

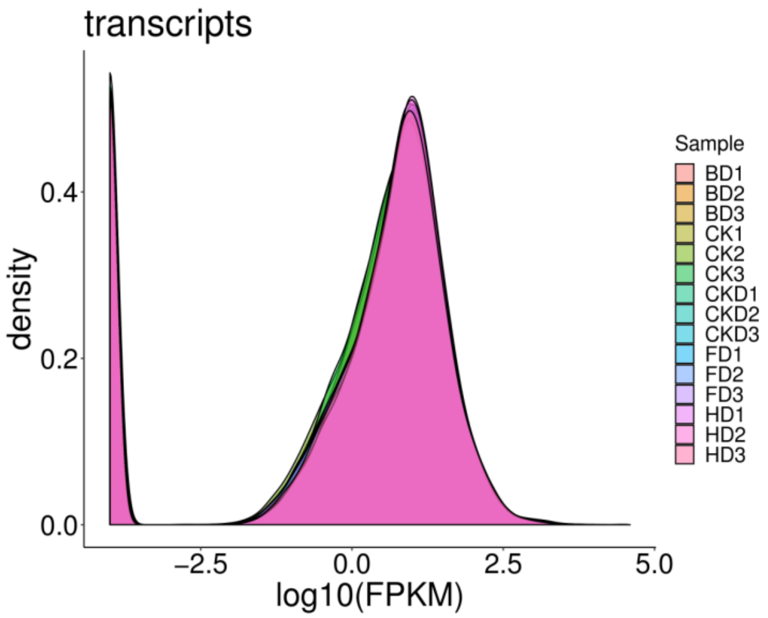

Figure S1. Expression density of transcripts in zucchini samples.

.

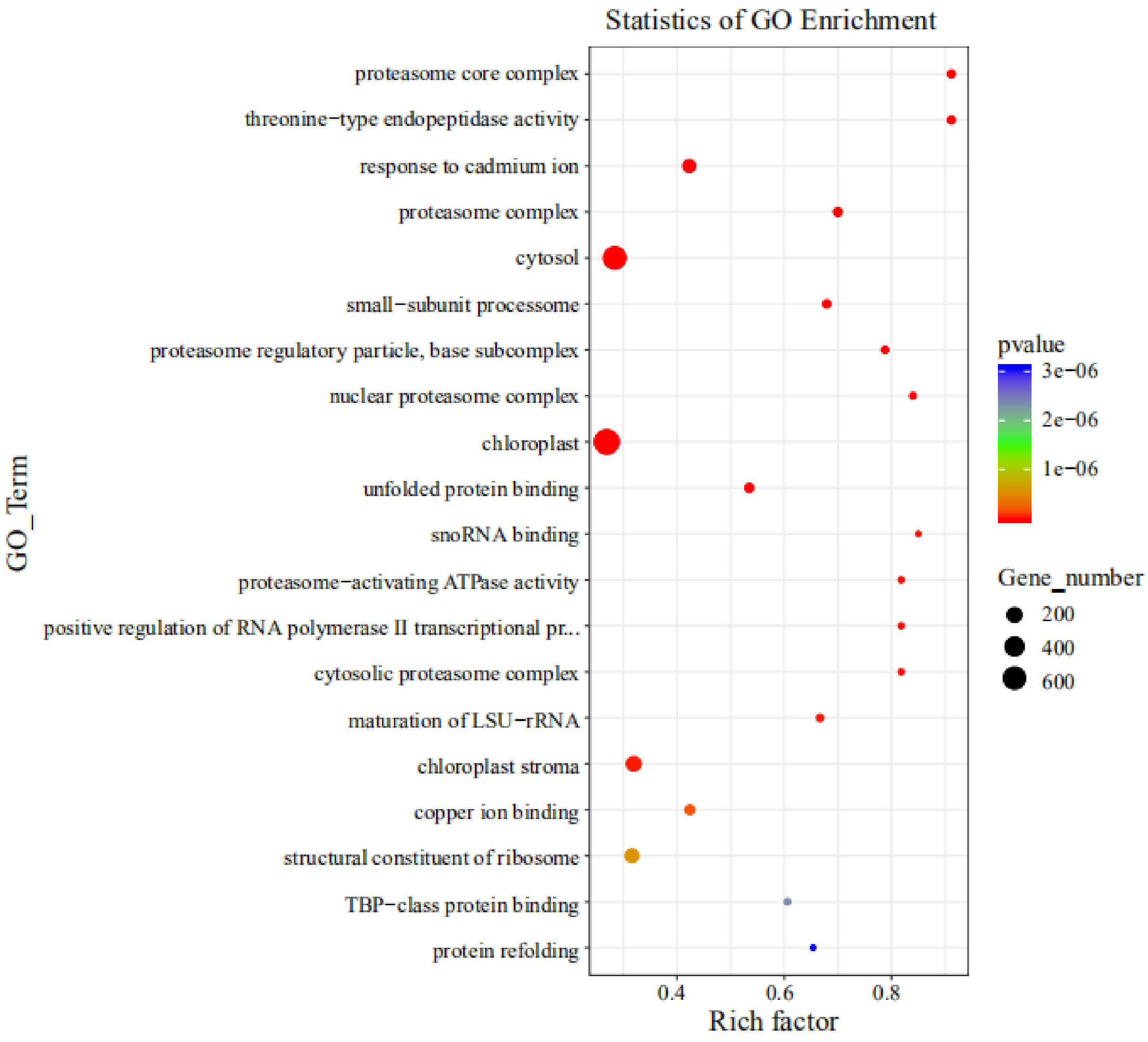

Figure S2. GO enrichment of DEGs in LT/CK comparison.

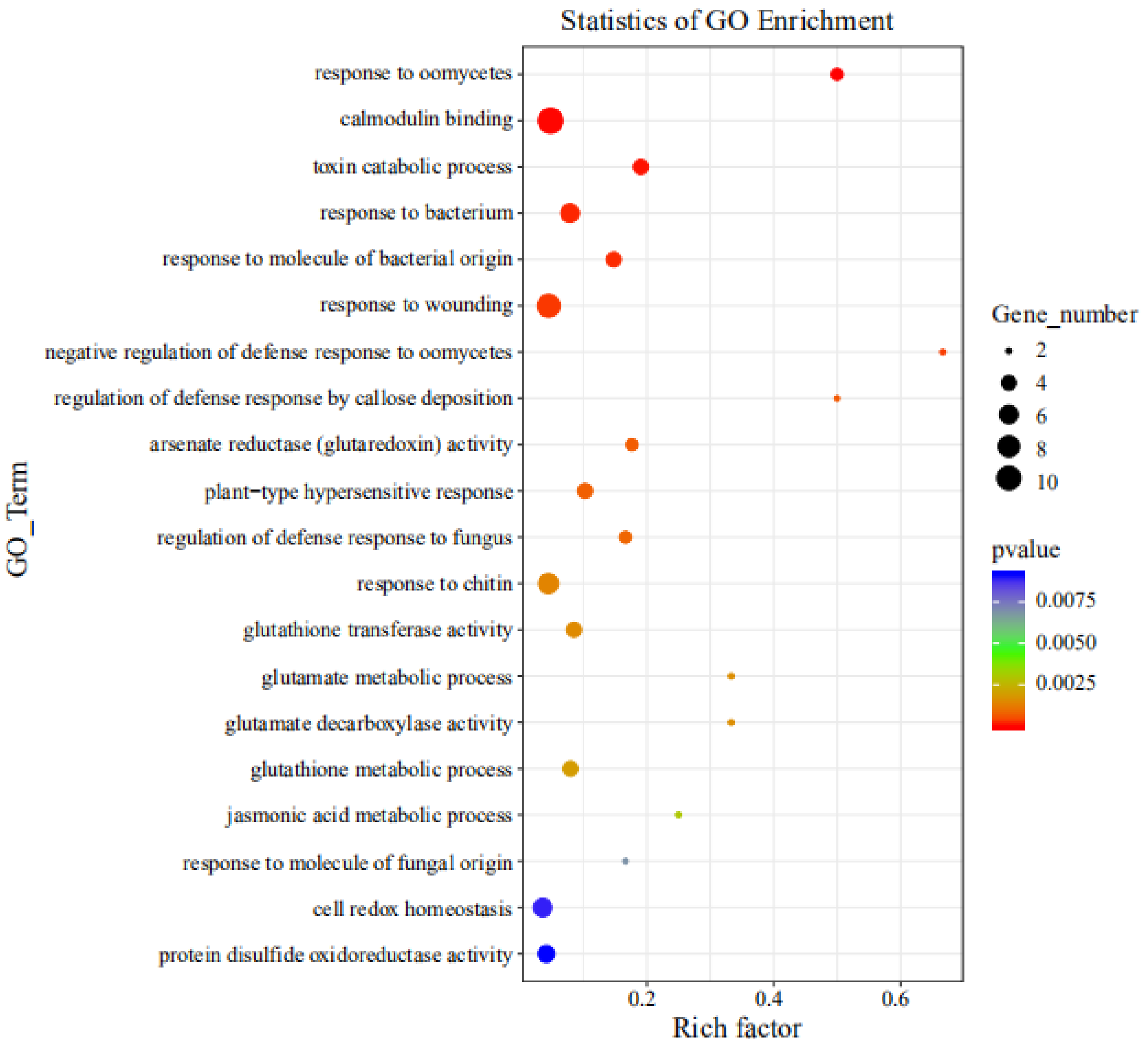

Figure S3. GO enrichment of DEGs in CHA/LT comparison.

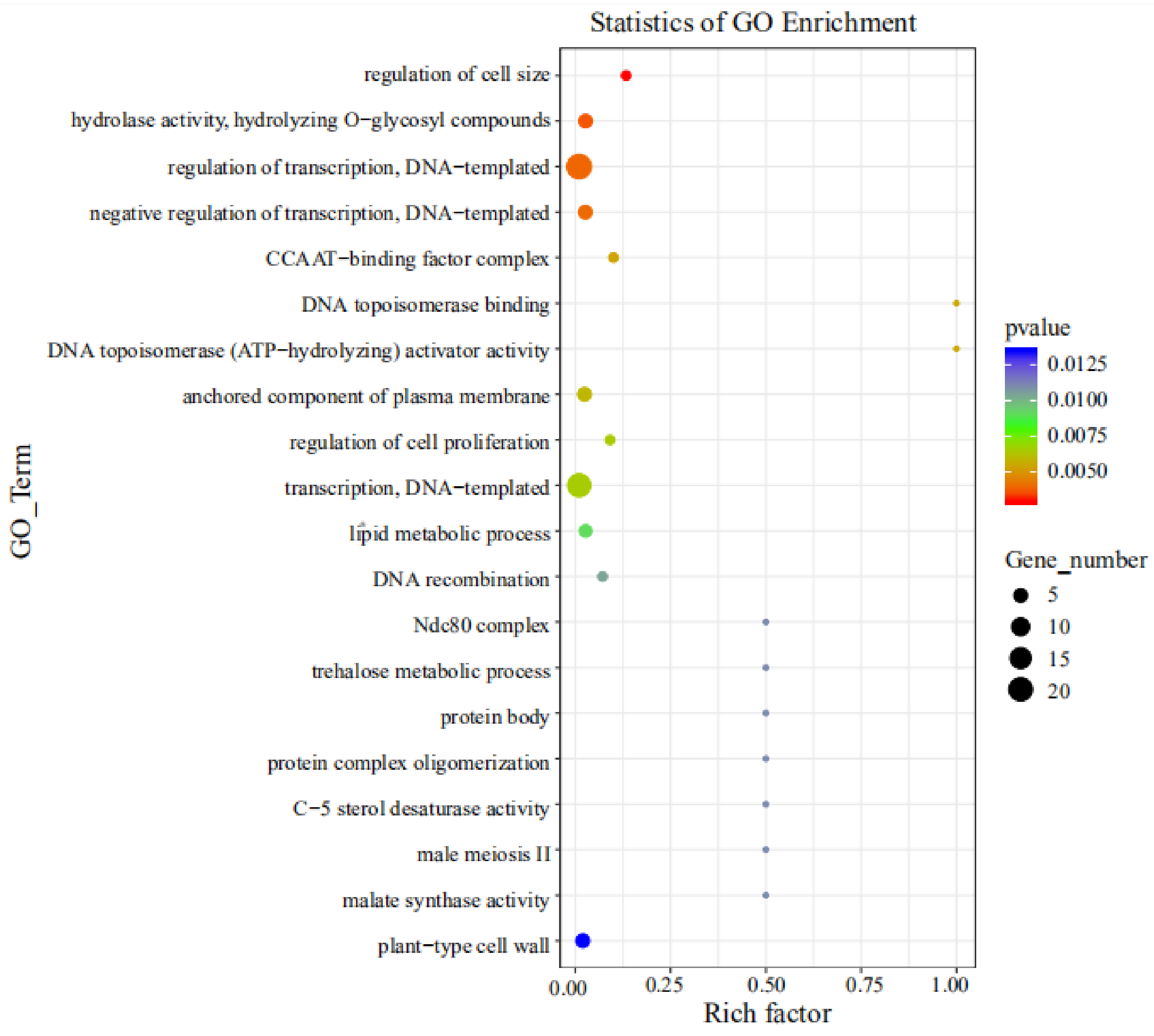

Figure S4. GO enrichment of DEGs in FA/LT comparison.

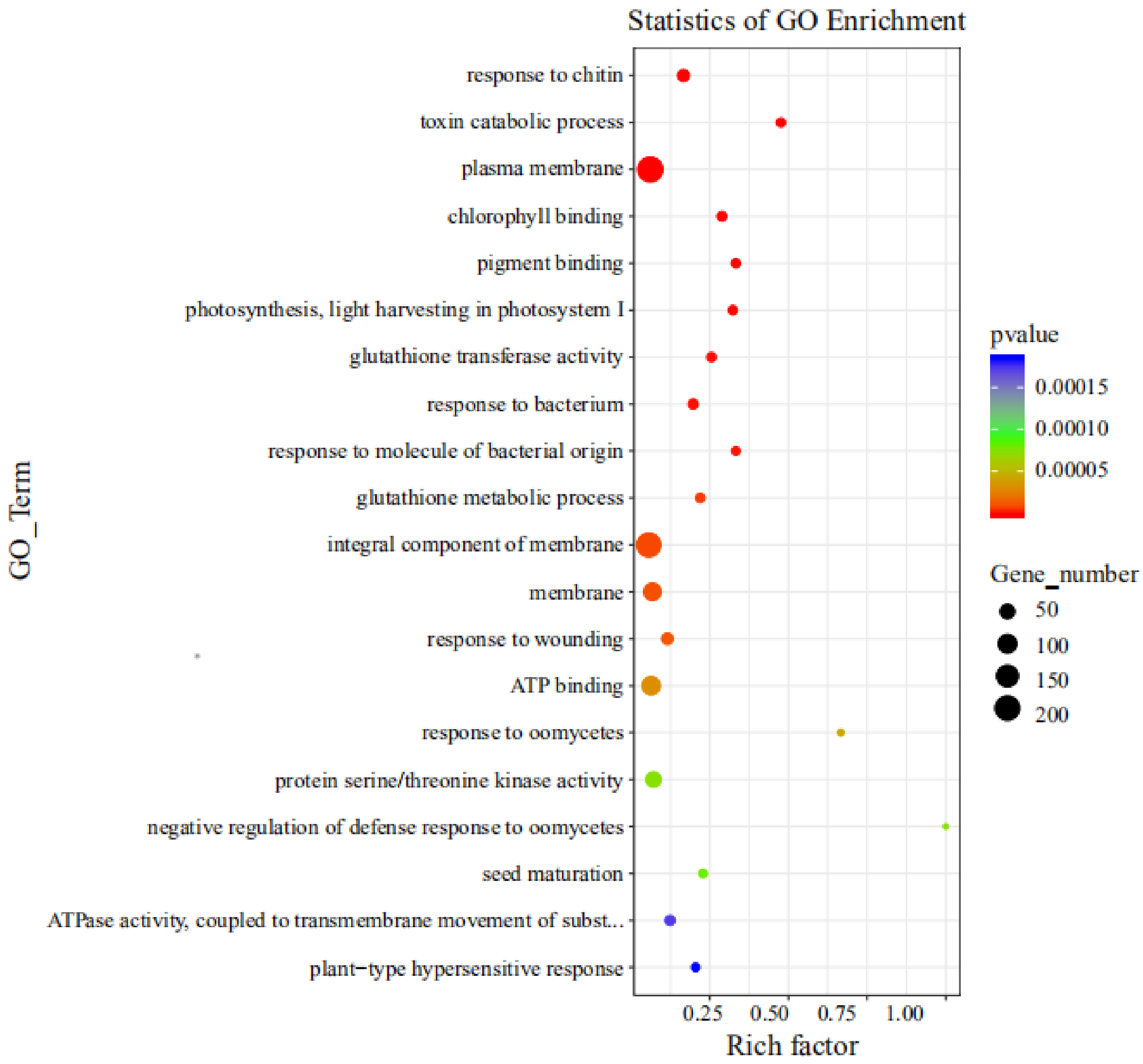

Figure S5. GO enrichment of DEGs in BHA/LT comparison.

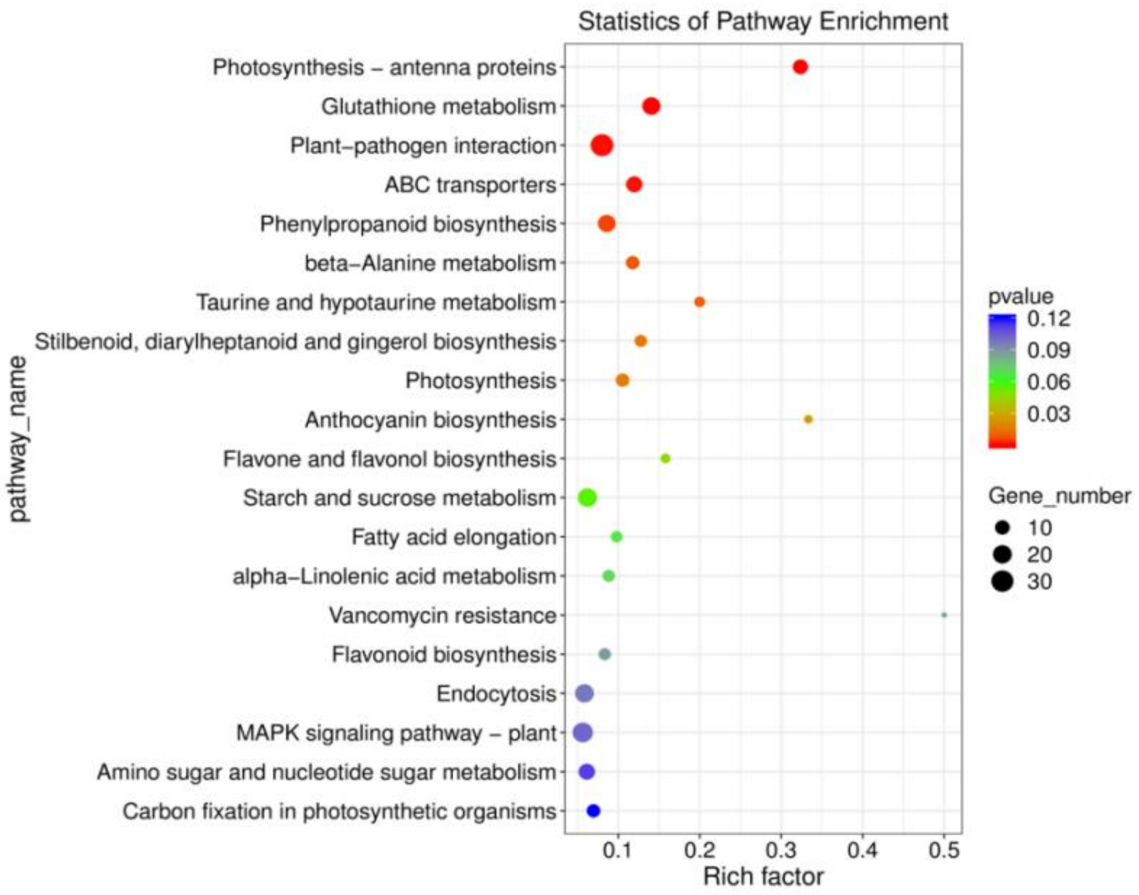

Figure S6. KEGG pathway of DEGs in LT/CK comparison.

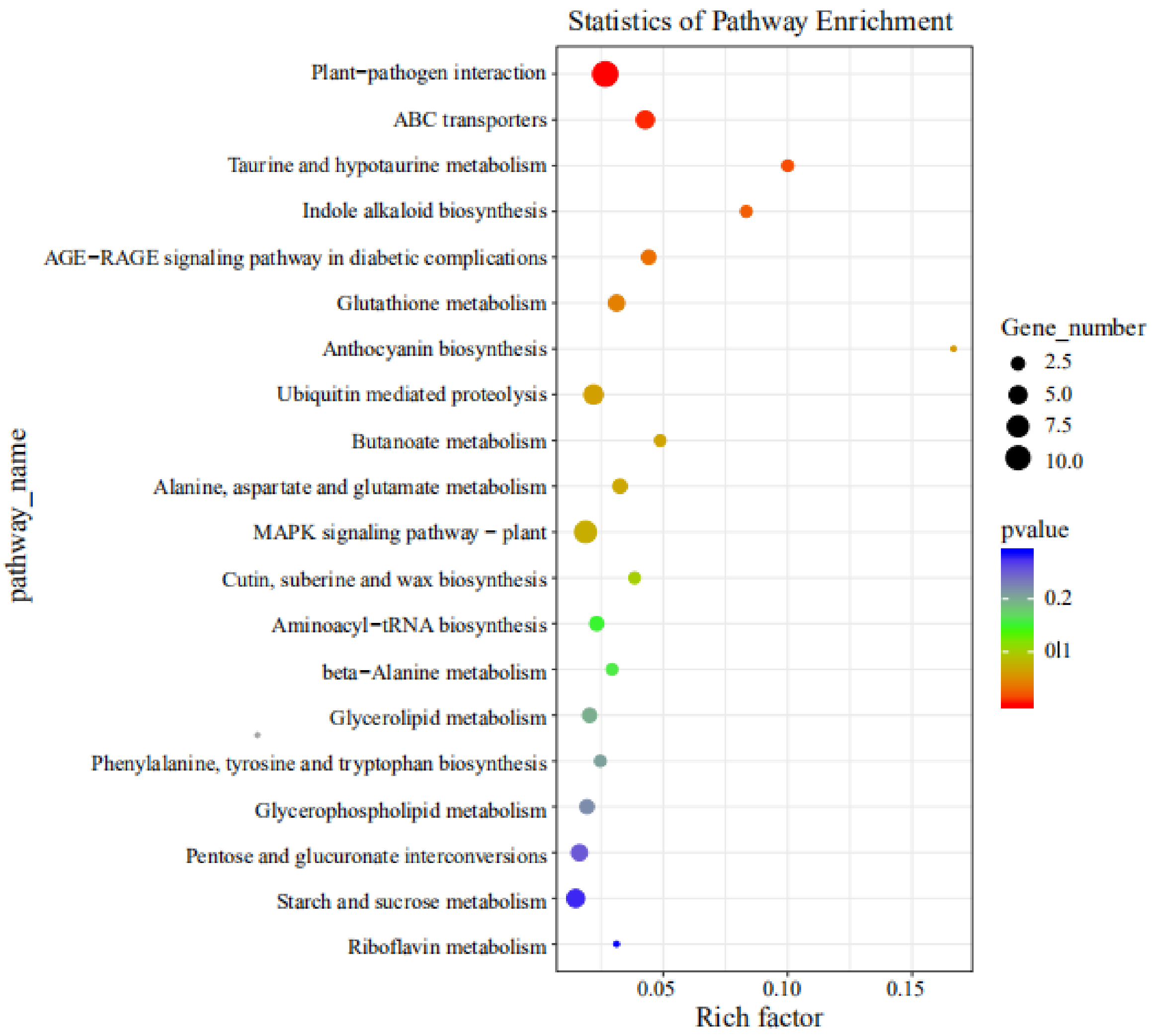

Figure S7. KEGG pathway of DEGs in CHA/LT comparison.

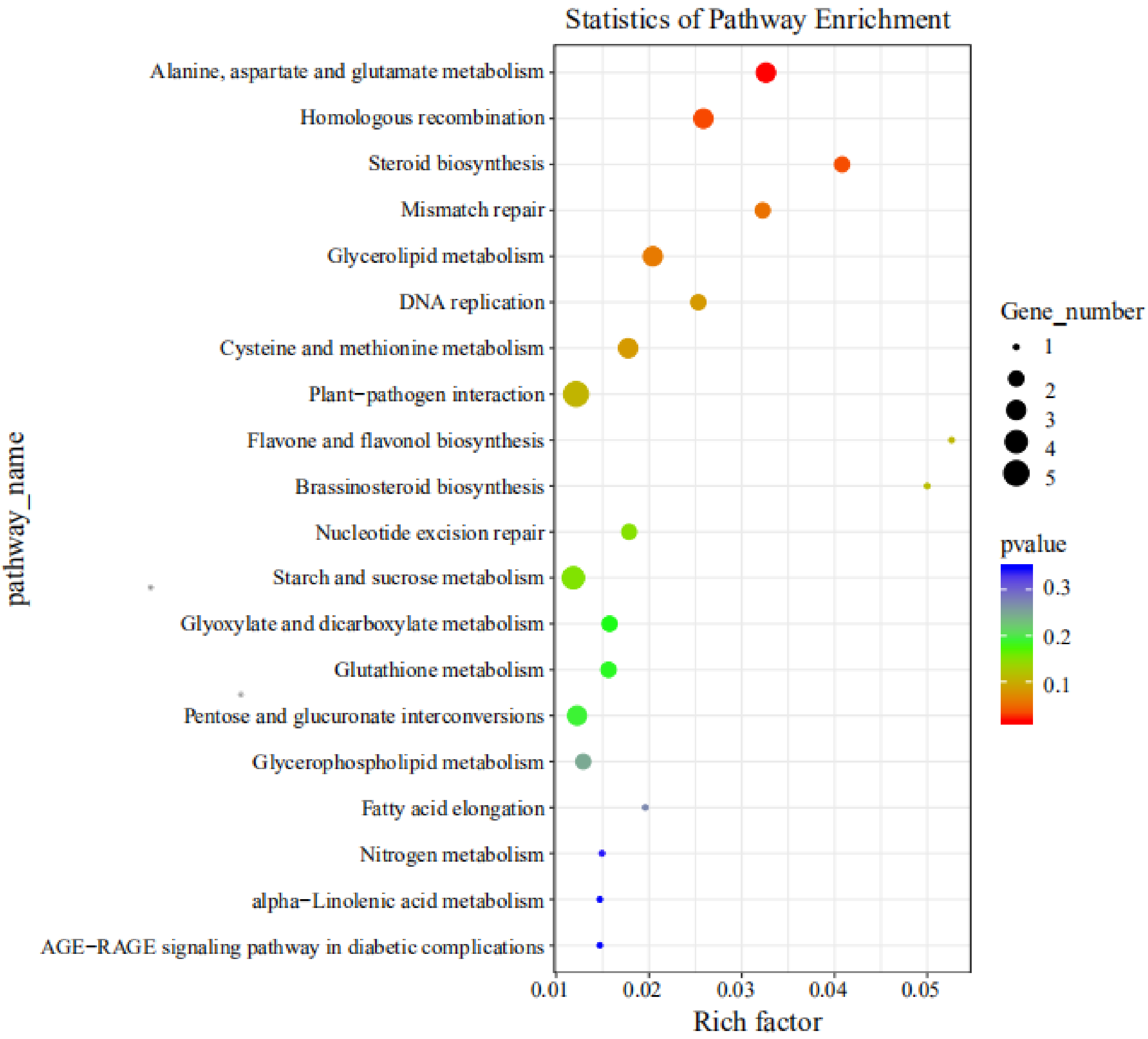

Figure S8. KEGG pathway of DEGs in FA/LT comparison.

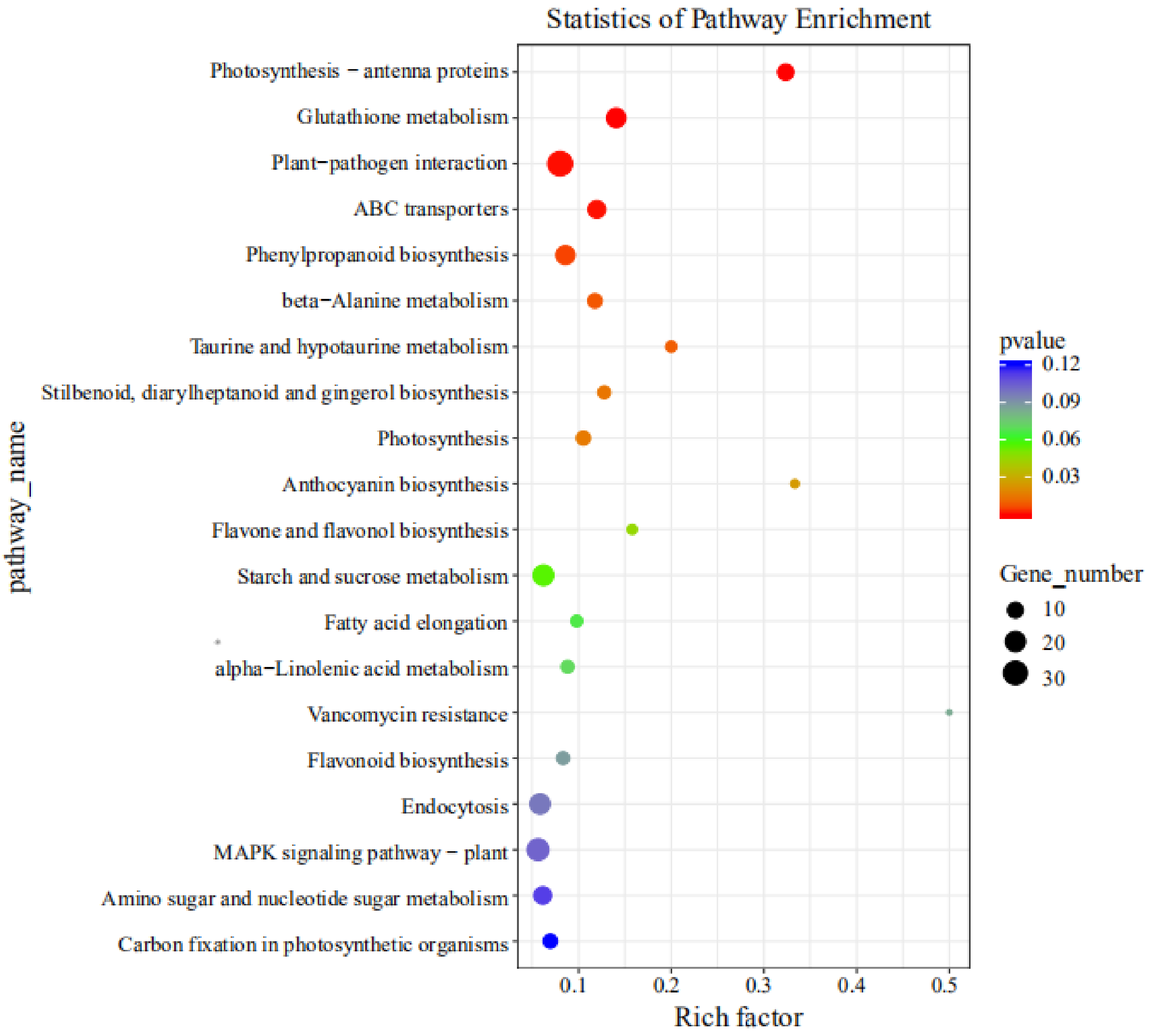

Figure S9. KEGG pathway of DEGs in BHA/LT comparison.

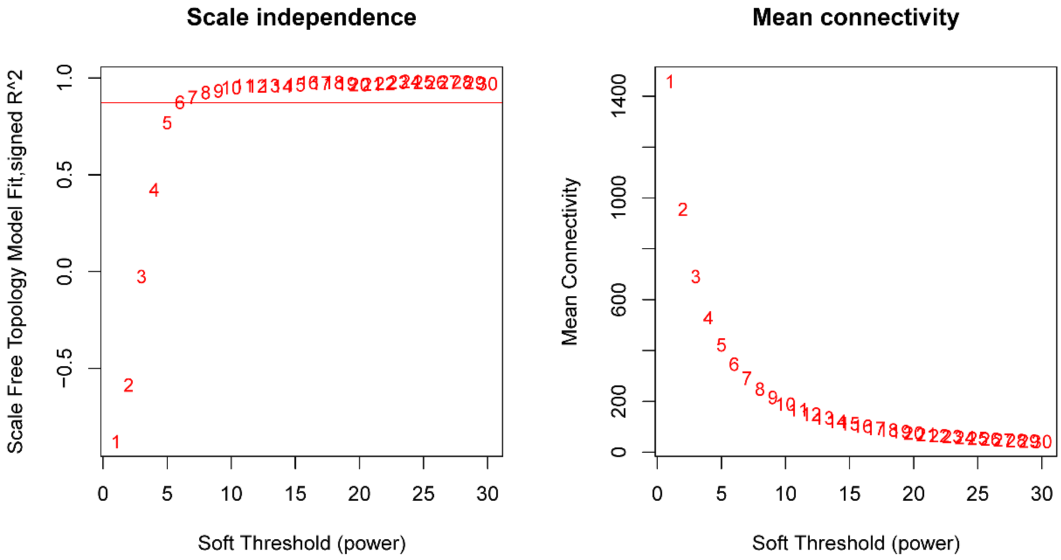

Figure S10. The soft threshold with scale independence **(*left*)** and mean connectivity **(*right*)** of WGCNA. The power value corresponding to the red line is the most appropriate power value of in the analysis.
